# Small interfering RNA-induced silencing lncRNA PVT1 inhibits atherosclerosis via inactivating the MAPK/NF-κB pathway

**DOI:** 10.18632/aging.203696

**Published:** 2021-11-13

**Authors:** Hong Du, Hui Zhang, Rong Yang, Li Qiao, Huiyu Shao, Xiaolin Zhang

**Affiliations:** 1 Department of Cardiology, Second Hospital of Hebei Medical University, Shijiazhaung 050000, Hebei, P.R. China

**Keywords:** atherosclerosis, human arterial vascular smooth muscle cells, MAPK/NF-κB pathway, PVT1, small interfering RNA

## Abstract

Atherosclerosis (AS) is a chronic disease of the arterial wall. The role of lncRNAs in AS has been acknowledged. This study investigated the role of lncRNA plasmacytoma variant translocation 1 (PVT1) in AS via the MAPK/NF-κB pathway. Serum samples were collected from AS and non-AS patients. Serum levels of PVT1, CRP, IL-6, IL-1β, and TNF-α were determined. AS mouse model was established and transfected with si-PVT1. Levels of TG, TC, HDL, LDL, MAPK, NF-κB, MMP-2, MMP-9, TIMP-1, and macrophage content were detected. Human arterial vascular smooth muscle cells (HA-VSMCs) induced by 50 mg/mL _ox_LDL were transfected with si-PVT1 or oe-PVT1 and added with MAPK inhibitor U0126. Viability, apoptosis, cell cycle, colony formation and DNA replication were assessed. Levels of apoptosis-related proteins were detected. Consequently, PVT1 was highly expressed in AS patients. Silencing PVT1 decreased levels of TG, TC, LDL, IL-6, IL-1β, TNF-α, MMP-2, MMP-9, CRP, TIMP-1, MAPK, and NF-κB, increased HDL, reduced atherosclerotic plaques and macrophage content in mice, inhibited viability, clones and EdU positive rates in HA-VSMCs, but promoted apoptosis and cell cycle arrest. Inhibition of MAPK/NF-κB pathway suppressed proliferation and promoted apoptosis of HA-VSMCs while PVT1 overexpression facilitated AS development. Briefly, silencing PVT1 inhibited AS development by downregulating MAPK/NF-κB pathway.

## INTRODUCTION

Atherosclerosis (AS) is manifested with chronic inflammation and dysfunction in the vascular system, which may cause severe consequences such as sudden cardiac death, myocardial infarction, peripheral thromboses and stroke [1]. Generally, endothelial dysfunction interacts with subendothelial lipoprotein retention, which takes place mainly in the intima of moderate arteries, particularly bifurcation points and arteriolar bifurcations, when blood flows are blocked [2, 3]. Besides, the treatment approaches for AS include limiting the hazard factors like hyperlipemia or high blood pressure and regulating inflammatory responses, among which regulating inflammatory response is still under development in the blood vessels [4]. Therefore, it is necessary to attach great importance to developing novel intervention strategies for AS.

Long noncoding RNAs (lncRNAs), normally defined as a set of transcripts with the length of more than 200 nucleotides without any protein-coding ability [5], are engaged in manipulating diverse cellular processes [6], vascular wall function, macrophage activation, lipid metabolism and inflammatory responses [7]. The lncRNA plasmacytoma variant translocation 1 (PVT1), a RNA gene of the lncRNA class, acts as a diagnostic marker for type 2 diabetes and a contributor to tumor development [8]. LncRNA PVT1 functions as an independent hazard factor for coronary AS progression and is highly expressed in AS patients [9], but the exact mechanism is still unclear. Recently, compelling evidence reveals novel approaches for AS based on the combination of lncRNA VINAS and the mitogen-activated protein kinase (MAPK) or nuclear factor-κB (NF-κB) pathways [10]. Activating the NF-κB or MAPK pathway is responsible to some extend for inflammatory responses in ischemic stroke [11]. Yousif et al*.* (2018) have verified the cardioprotective effects exerted by the inactivation of the p38-MAPK/NF-κB pathways with the involvement of Irbesartan against polymicrobial sepsis, a systemic inflammatory response usually correlated with several organ failures, possibly by attenuating myocardial dysfunction and reducing pro-inflammatory cytokines [12]. In addition, Pan (2017) has confirmed the critical function of lncRNA H19 in regulating AS through the MAPK and NF-κB pathways [13]. From above mentioned findings, it is reasonable to hypothesize the implication of lncRNA PVT1 and the MAPK/NF-κB signaling pathway in AS progression. Thus, we implemented a study to explore how lncRNA PVT1 acts on AS by regulating the MAPK/NF-κB pathway with the expectation to seek out novel clinical value for AS patients.

## RESULTS

### PVT1 is highly expressed in AS patients

**Figure 1:**
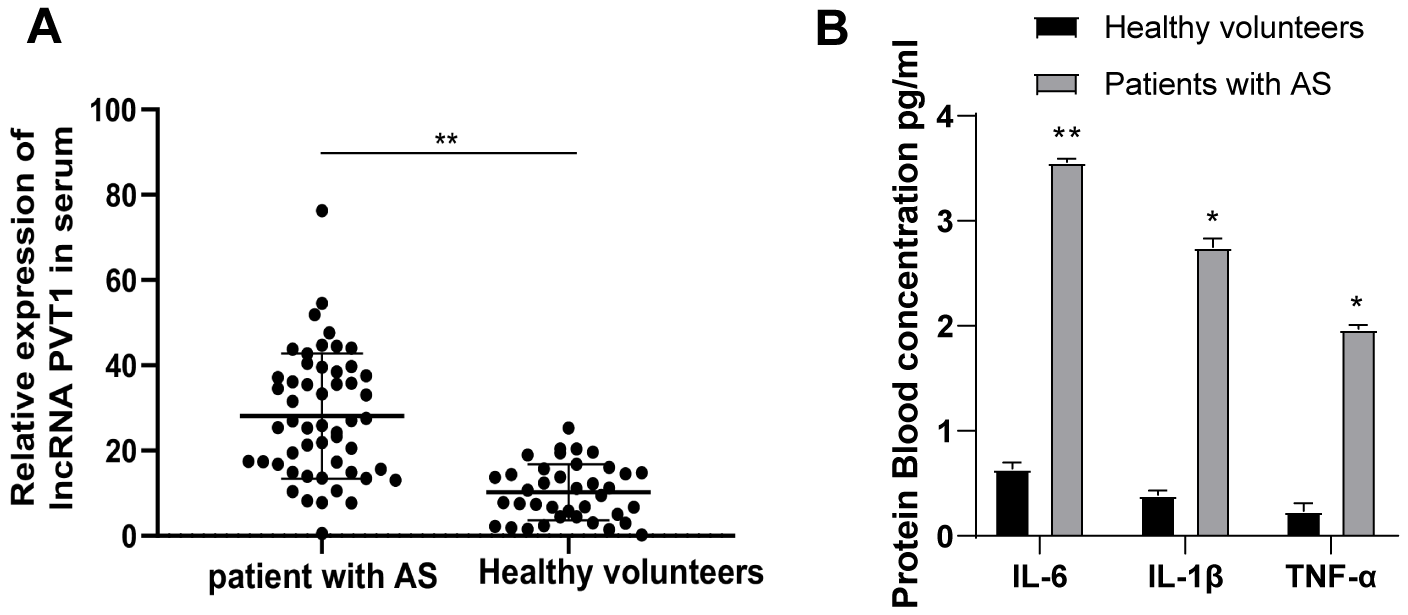
**PVT1 is highly expressed in AS patients.** (**A**) Relative PVT1 expression in serum of AS patients and healthy controls detected by qRT-PCR; (**B**) Levels of IL-6, IL-1β and TNF-α in serum of AS patients detected by ELISA. * *p <* 0.05, ** *p <* 0.01. PVT1, plasmacytoma variant translocation 1; AS, atherosclerosis; CRP, C-reactive protein; IL, interleukin; TNF-α, tumor necrosis factor-alpha.

We first detected PVT1 expression in the serum of 52 AS patients and 32 healthy people by qRT-PCR. It was found that PVT1 expression in AS patients was significantly increased relative to that in healthy people (Figure 1A) (*p* < 0.05). Besides, AS is regarded as an inflammatory condition in the arterial wall. So, we measured the levels of IL-6, IL-1β, and TNF-α in the serum of AS patients and healthy people with ELISA kits. The results demonstrated that levels of IL-6, IL-1β and TNF-α were substantially higher in AS patients than those in healthy people (Figure 1B) (*p* < 0.05).

### Silencing PVT1 reduces AS damage in ApoE-/-mice

**Figure 2:**
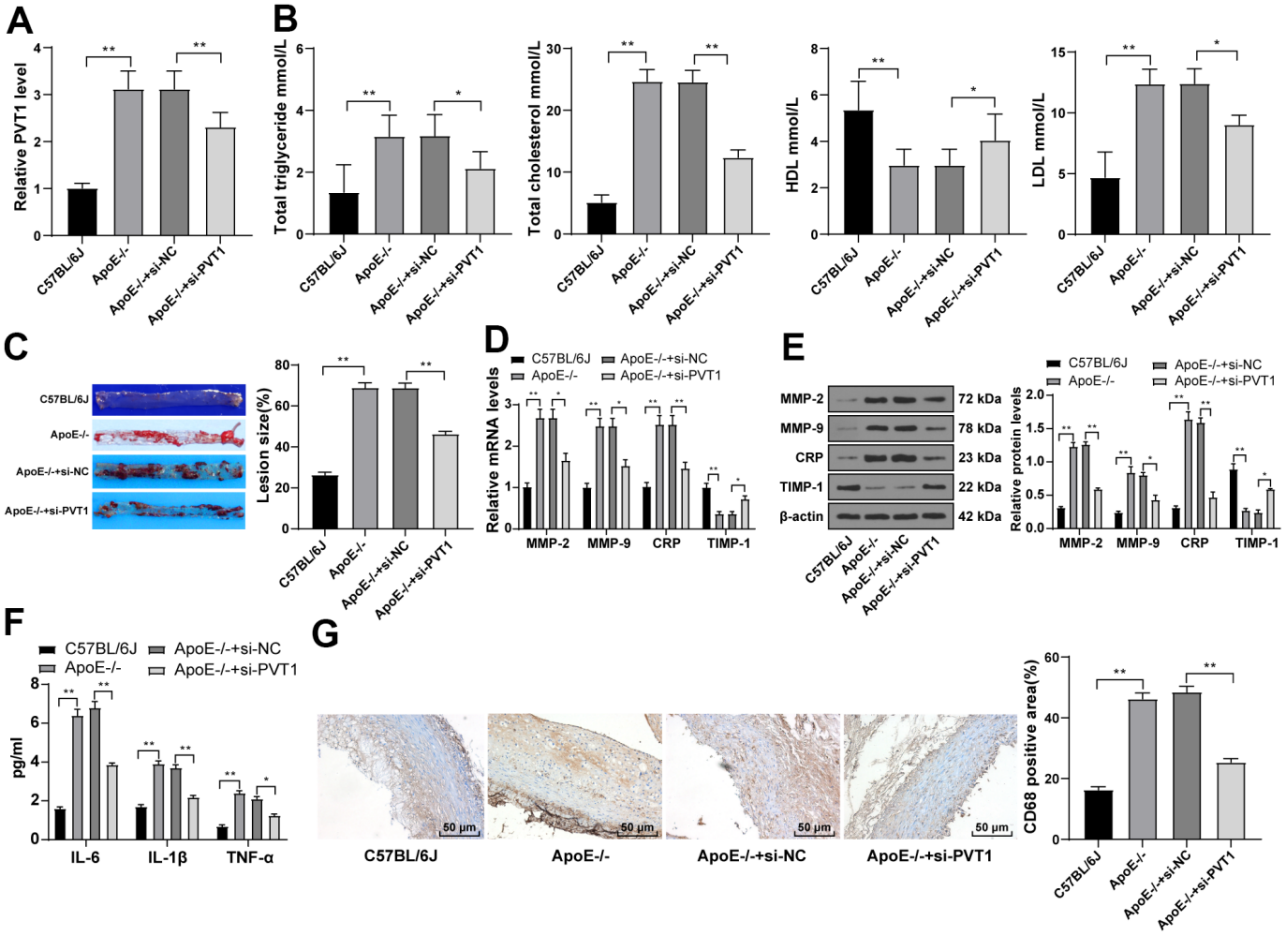
**Silencing PVT1 reduces AS damage in ApoE-/- mice.** ApoE-/- mice were fed with high-fat diet for 12 weeks. (**A**) Relative PVT1 expression in mice detected by qRT-PCR; (**B**) Levels of TG, TC, HDL, and LDL detected by ELISA; (**C**) Lipid accumulation on the aortic wall detected by oil red O staining; (**D**) mRNA levels of MMP-2, MMP-9, CRP, and TIMP-1 detected by qRT-PCR; (**E**) Protein levels of MMP-2, MMP-9, CRP and TIMP-1 detected by Western blot; (**F**) Levels of IL-6, IL-1β, and TNF-α detected by ELISA; (**G**) CD68 positive cells detected by immunohistochemistry. N = 6. Data were presented as mean ± standard deviation. Comparisons among multi-groups were analyzed using one-way ANOVA, followed by Tukey’s multiple comparisons test. * *p <* 0.05, ** *p <* 0.01. PVT1, plasmacytoma variant translocation 1; AS, atherosclerosis; ApoE-/-, apolipoprotein E knockout; TG, total triglyceride; TC, total cholesterol; HDL, high density lipoprotein; LDL, low density lipoprotein; MMP, matrix metalloproteinase; TIMP-1, tissue inhibitor of metalloproteinase-1; CRP, C-reactive protein; IL, interleukin; TNF-α, tumor necrosis factor-alpha.

LncRNA PVT1 was reported to serve as an independent hazard factor in AS development and highly expressed in AS patients [9]. Therefore, the expression of PVT1 in mice was determined by qRT-PCR, which revealed that PVT1 expression in ApoE-/-mice was greatly elevated relative to that in wild C57BL/6J mice fed with normal diet (Figure 2A) (p < 0.05). After silencing PVT1, the serum levels of TG, TC, and LDL were significantly decreased and the serum level of HDL was elevated in ApoE-/-mice (Figure 2B) (p < 0.05). The oil red O staining exhibited that the number and size of AS plaques in ApoE-/-mice were evidently decreased after silencing PVT1 (Figure 2C) (p < 0.05). The results of qRT-PCR and Western blot analysis presented that levels of matrix metalloproteinase 2 (MMP-2), MMP-9, and CRP in AS plaques in ApoE-/-mice were notably decreased while levels of tissue inhibitor of metalloproteinase-1 (TIMP-1) were increased after silencing PVT1 (Figure 2D–2E) (all p < 0.05). ELISA results demonstrated that the levels of IL-6, IL-1β and TNF-α in ApoE-/-mice were obviously decreased (Figure 2F) (p < 0.05), and immunohistochemistry showed macrophages in ApoE-/-mice were significantly reduced after silencing PVT1 (Figure 2G) (p < 0.05). Briefly, silencing PVT1 reduces AS damage in ApoE-/-mice.

### PVT1 is highly expressed in _ox_LDL-treated HA-VSMCs

**Figure 3:**
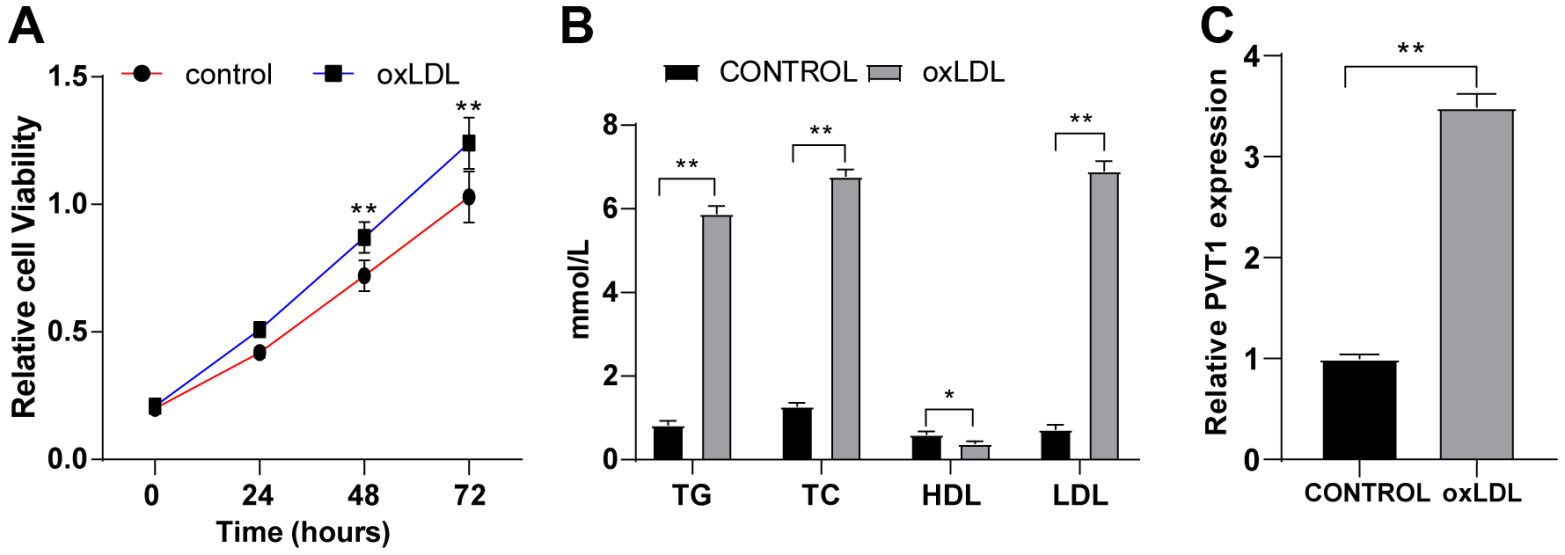
**PVT1 is highly expressed in _ox_LDL-treated HA-VSMCs.** HA-VSMCs were treated with 50 mg/mL _ox_LDL for 24 hr. (**A**) Cell viability detected by MTT assay; (**B**) Levels of TG, TC, HDL, and LDL detected by ELISA; (**C**) LncRNA PVT1 relative expression detected by qRT-PCR. N = 3. Data were presented as mean ± standard deviation. Pairwise comparisons were analyzed using independent sample *t* test. * *p* < 0.05, ** *p* < 0.01. PVT1, plasmacytoma variant translocation 1; _ox_LDL, oxidative low density lipoprotein; HA-VSMCs, Human arterial vascular smooth muscle cells.

HA-VSMCs were treated for 24 h with 50 mg/mL _ox_LDL. MTT assay showed enhanced viability of HA-VSMCs after _ox_LDL treatment (Figure 3A) (*p* < 0.05). ELISA showed elevated levels of TG, TC and LDL and diminished HDL level in HA-VSMCs after _ox_LDL treatment (Figure 3B) (*p* < 0.05), indicating the presence of lipid metabolism disorder in HA-VSMCs after _ox_LDL treatment and successful establishment of AS cell model. The expression of PVT1 in _ox_LDL-treated HA-VSMCs was determined by qRT-PCR. We found highly expressed PVT1 in _ox_LDL-treated HA-VSMCs compared to that in untreated cells (Figure 3C) (*p* < 0.05). These results elicited that PVT1 is highly expressed in _ox_LDL-treated HA-VSMCs.

### Silencing PVT1 inhibits the viability and proliferation of HA-VSMCs

**Figure 4:**
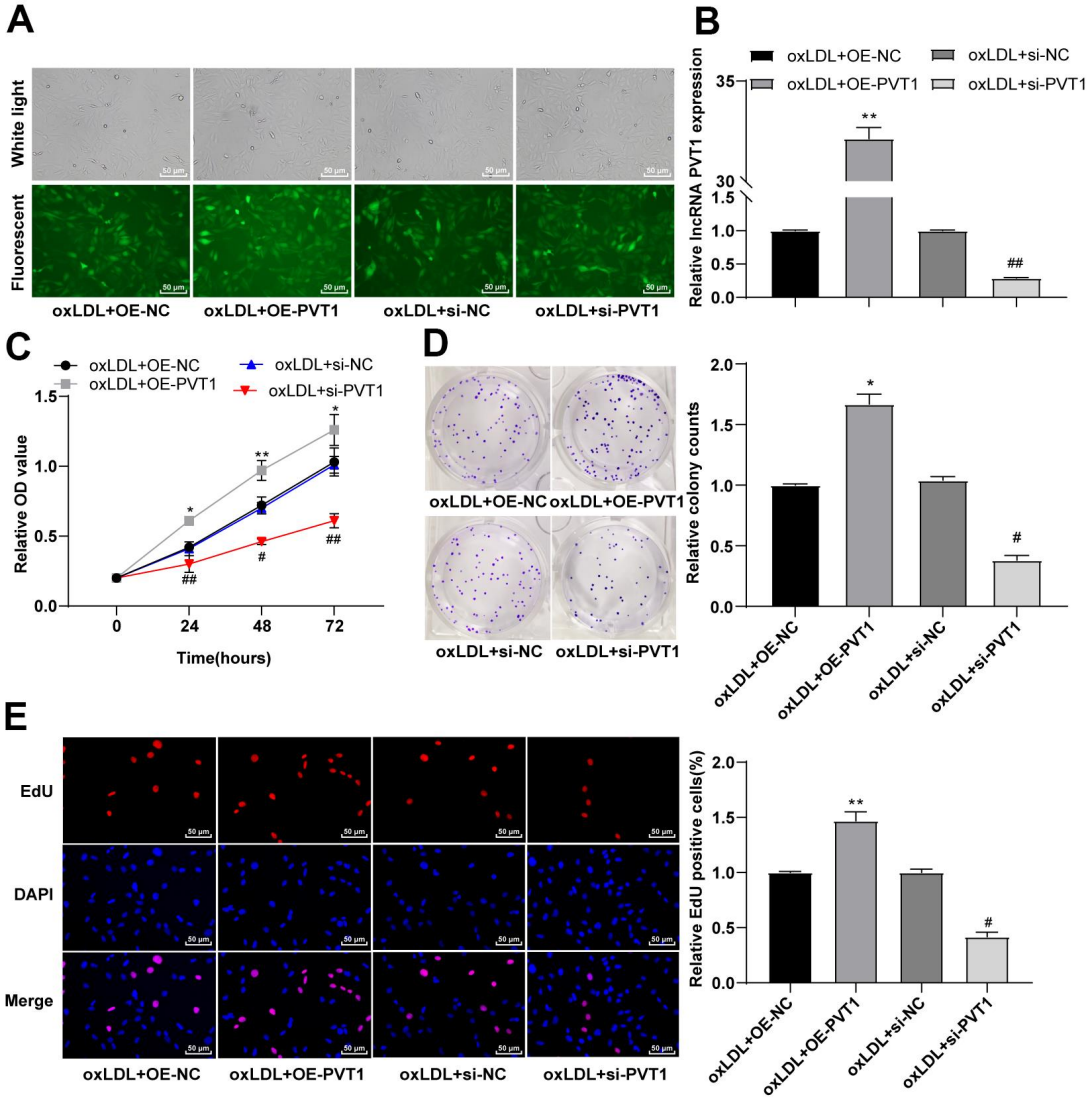
**Silencing PVT1 induced by siRNA inhibits the activity and proliferation of HA-VSMCs.** (**A**) Fluorescence of GFP ensures the recombinant plasmid was transfected properly; (**B**) Relative expression of PVT1 detected by qRT-PCR; (**C**) Cell viability measured by MTT assay; (**D**) Cell colony formation ability detected by colony formation assay; (**E**) DNA replication activity detected by EdU assay. Compared to the oe-NC group, * *p* < 0.05, ** *p* < 0.01; compared to the si-NC group, # *p* < 0.05, ## *p* < 0.01. PVT1, plasmacytoma variant translocation 1; HA-VSMCs, Human arterial vascular smooth muscle cells; oe, overexpression; NC, negative control.

The abnormal proliferation of VSMCs contributes to plaque formation [14–17]. To investigate the involvement of lncRNA PVT1 in the abnormal proliferation of oxLDL-induced VSMCs, oxLDL-induced VSMCs were transfected with oe-NC, oe-PVT1, si-NC and si-PVT1, respectively. Green fluorescence was observed after 48 h of transfection (Figure 4A) (p < 0.05), and the expression of PVT1 was verified by qRT-PCR (Figure 4B) (p < 0.05). MTT assay indicated that the cell viability was notably facilitated at 24 h, 48 h and 72 h after PVT1 overexpression, and was obviously decreased after silencing PVT1 (Figure 4C) (*p* < 0.05). Colony formation assay showed clones were greatly increased after PVT1 overexpression, and were significantly decreased after silencing PVT1 (Figure 4D) (*p* < 0.05). EdU assay suggested that the positive rate of EdU was markedly augmented after PVT1 overexpression, and was decreased after silencing PVT1 (Figure 4E) (all *p* < 0.05). Altogether, silencing PVT1 inhibits the viability and proliferation of VSMCs.

### Silencing PVT1 promotes apoptosis and cell cycle arrest of HA-VSMCs

**Figure 5:**
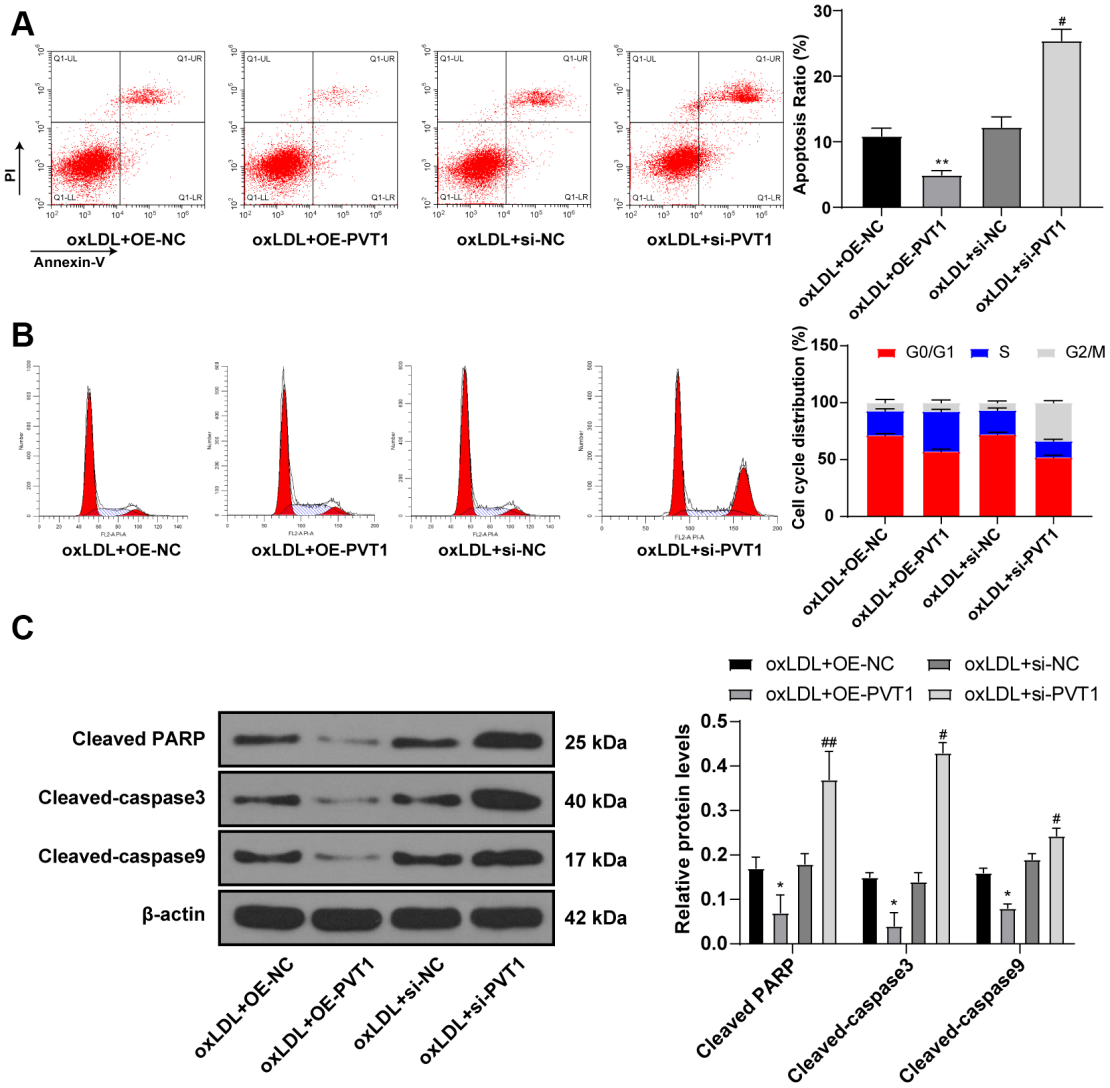
**Silencing PVT1 promotes apoptosis and cell cycle arrest of HA-VSMC.** (**A**) Relative apoptosis rate of HA-VSMCs detected by flow cytometry; (**B**) Relative cell cycle distribution of HA-VSMCs; (**C**) Relative levels of apoptosis-related proteins detected by Western blot; compared to the oe-NC group, *p < 0.05, ** *p* < 0.01; compared to the si-NC group, # *p* < 0.05, ## *p* < 0.01. PVT1, plasmacytoma variant translocation 1; HA-VSMCs, human arterial vascular smooth muscle cells; oe, overexpression; NC, negative control.

Furthermore, flow cytometry detected cell apoptosis and cell cycle, and showed less apoptotic cells with a large number of cells in S phase after PVT1 overexpression while apoptotic cells were significantly increased with diminished S phase cells and augmented G2/M phase cells after silencing PVT1 (Figure 5A, 5B) (all *p* < 0.05). Western blot analysis was used to detect the levels of cell apoptotic-related proteins and the results showed decreased levels of cleaved caspase-3, poly(ADP-ribose) polymerase (PARP), and cleaved caspase-9 after PVT1 overexpression and increased levels of cleaved caspase-3, PARP, and cleaved caspase-9 after silencing PVT1 (Figure 5C) (*p* < 0.05). Collectively, silencing PVT1 promotes apoptosis and cell cycle arrest of VSMCs.

### Silencing PVT1 downregulates the MAPK/NF-κB signaling pathway in _ox_LDL-treated HA-VSMCs and AS mice

**Figure 6:**
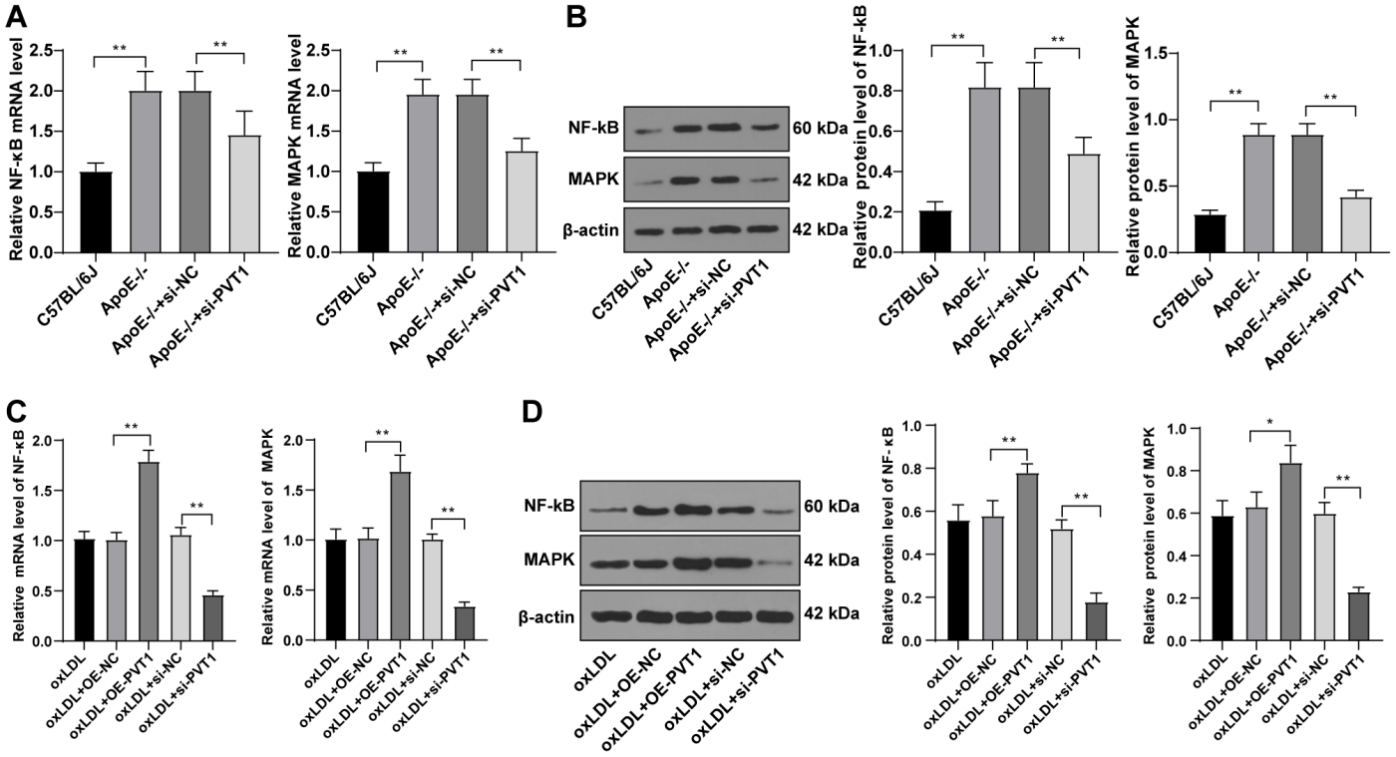
**Silencing PVT1 downregulates the MAPK/NF-κB signaling pathway in _ox_LDL-induced HA-VSMCs and AS mice.** (**A**) mRNA expression of MAPK and NF-κB in AS mice detected by qRT-PCR; (**B**) Protein levels of MAPK and NF-κB in AS mice detected by Western blot; (**C**) mRNA expression of MAPK and NF-κB in _ox_LDL-treated HA-VSMCs detected by qRT-PCR; (**D**) Protein levels of MAPK and NF-κB in _ox_LDL treated HA-VSMCs detected by Western blot. Compared to the oe-NC group, * *p* < 0.05, ** *p* < 0.01; compared to the si-NC group, # *p* < 0.05, ## *p* < 0.01. PVT1, plasmacytoma variant translocation 1; MAPK, mitogen-activated protein kinase; NF-κB, Nuclear factor-kappa B; _ox_LDL, oxidative low density lipoprotein; AS, atherosclerosis; HA-VSMCs, Human arterial vascular smooth muscle cells; oe, overexpression; NC, negative control.

PVT1 upregulation activates the MAPK/NF-κB pathway and promotes secretion of inflammatory factors while silencing PVT1 inhibits the the MAPK/ NF-κB pathway [18]. As the MAPK/NF-κB pathway is important to promote the secretion of IL-1, IL-6, IL-8, and TNF-α and could sever as a transcriptional regulator for recruiting macrophages and monocytes, we detected levels of MAPK and NF-κB in AS mice. The results displayed decreased levels of MAPK and NF-κB in ApoE-/- mice (Figure 6A, 6B) (all *p* < 0.05).

Subsequently, the mRNA and protein levels of MAPK and NF-κB in oxLDL-treated HA-VSMCs were determined by qRT-PCR and Western blot analysis, and elevated levels of MAPK and NF-κB (Figure 6C) (p < 0.05), and decreased levels of MAPK and NF-κB were observed in the oxLDL + si-PVT1-treated cells (Figure 6D) (p < 0.05). These results confirmed that silencing PVT1 downregulates the MAPK/NF-κB signaling pathway in oxLDL-treated HA-VSMCs and AS mice.

### Silencing PVT1 suppresses AS progression via downregulating the MAPK/NF-κB pathway

**Figure 7:**
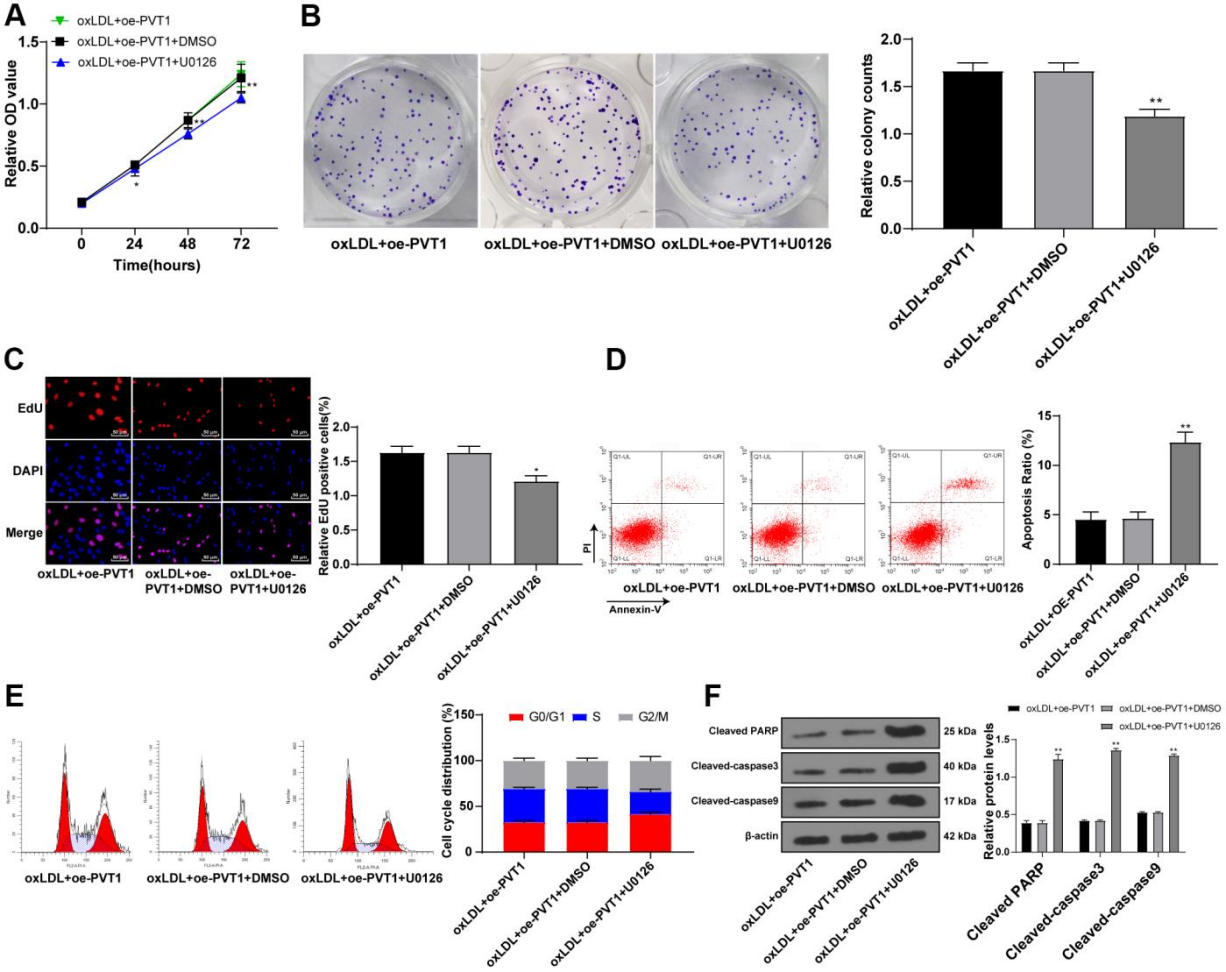
**Silencing PVT1 suppresses AS progression via downregulating the MAPK/NF-κB signaling pathway.** (**A**) Cell viability measured by MTT assay; (**B**) Relative cell clones measured by colony experiment; (**C**) Relative EdU positive rates detected by EdU assay; (**D**) Relative apoptosis ratio detected by flow cytometry; (**E**) Relative cell cycle distribution; (**F**) Relative protein levels of cleaved caspase-3, PARP and cleaved caspase-9 detected by Western blot. Compared to the PVTI-DMSO group, * *p* < 0.05, ** *p* < 0.01. PVT1, plasmacytoma variant translocation 1; AS, atherosclerosis; MAPK, mitogen-activated protein kinase; NF-κB, Nuclear factor-kappa B; OD, optical value; EdU, 5-ethynyl-2’-deoxyuridine; PARP, PARP, poly(ADP-ribose) polymerase; DMSO, dimethyl sulfoxide.

The aforementioned data unraveled that silencing PVT1 downregulated the MAPK/NF-κB pathway. Moreover, the inactivation of the MAPK/NF-κB pathway suppresses AS development [19–21]. To further confirm the effect of PVT1 on AS via the MAPK/NF-κB pathway, we added a specific inhibitor of MAPK, U0126, to oxLDL-treated HA-VSMCs after PVT1 overexpression. The results showed that the cell viability, cell clones and EdU positive rates of HA-VSMCs were noticeably downregulated afterwards (Figure 7A–7C) (all p < 0.05). The apoptotic cells and G2/M-phase cells were augmented while S-phase cells were reduced (Figure 7D, 7E) (p < 0.05). Western blot analysis exhibited an increase in levels of cleaved caspase-3, PARP and cleaved caspase-9 (Figure 7F) (all p < 0.05). The abovementioned results ascertained that silencing PVT1 suppresses AS development by downregulating the MAPK/NF-κB pathway.

## DISCUSSION

Recognized as a common malignant disease of the vessel wall and a chronic inflammatory process, AS contributes to increased morbidity and mortality rates for patients with cardiovascular and cerebrovascular diseases, and is still a global burden with increasing incidence in developing countries [22]. LncRNAs exert essential functions on vascular inflammatory response and metabolism in endothelial cells and macrophages, suggestive of the possible effects of lncRNAs on AS development [23]. Thus, we studied the mechanism of lncRNA PVT1 in AS progression with the MAPK/NF-κB pathway involved. Collectively, we found that silencing PVT1 suppressed AS progression by downregulating the MAPK/NF-κB signaling pathway.

The first chief result of the current study was that lncRNA PVT1 was highly expressed in the serum of AS patients, ApoE-/-mice and oxLDL-treated HA-VSMCs, which was in consistency with a previous study [9]. The unfavorable role of PVT1 was further identified in other diseases. For instance, increased expression of PVT1 was found in hippocampus tissues of epileptic rats and was associated with neuronal loss and inflammation [24].

PVT1 expression was increased in acute ischemic stroke patients [25], atrial fibrosis patients [26] and patients with cardiomyocyte toxicity [27]. Besides, we found silencing PVT1 reduced levels of TG, TC, HDL and LDL, MMP-2, MMP-9, CRP, and levels of inflammation-related factors, but increased TIMP expression. AS is considered as an inflammatory disease and lipid metabolism disorder [28]. MMPs are related to AS progression [29]. CRP, the representative acute phase protein, is sensitive for inflammation and tissue damage, and its elevated plasma level is correlated with increased risk of cardiovascular diseases [30]. si-PVT1 diminished levels of TNF-α, IL-6 and IL-1β in lipopolysaccharide-induced HK-2 cells, thus alleviating inflammation and acute kidney injury [31]. Silencing PVT1 downregulates MMP-1 and MMP-9 protein expression and upregulates TIMP-1 in ApoE-/- mice [32]. In addition, a recent study noted that knockdown of PVT1 decreased levels of MMP-2 and MMP-9, augmented TIMP-1 expression, and suppressed serum levels of TNF-α, IL-1β, and IL-6 in VAMC and ApoE-/- mice of abdominal aortic aneurysm model [32]. Altogether, silencing PVT1 could attenuate AS damage.

Moreover, the data obtained in this study confirmed that silencing PVT1 inhibited HA-VSMC viability, proliferation and promoted apoptosis and cell cycle arrest, presenting by elevated cleaved caspase-3, PARP, and cleaved caspase-9. The cleavage and activation of caspase-3, known as a key effector protease, in apoptotic process cleaves PARP, which serves as an apoptotic biomarker [33]. Silencing PVT1 increases expressions of Bax and cleaved caspase-3 and thus inhibits apoptosis in diabetic nephropathy [34]. LncRNA PVT1 promoted MMP production and facilitated extracellular matrix degradation, leading to VSMC apoptosis and the formation of abdominal aortic aneurysm [32]. PVT1 depletion impaired cell growth and migration ability of vascular endothelial cells [35]. PVT1 knockdown could induce apoptosis in lipopolysaccharide-induced H9c2 cells, through up-regulating Bax and caspase-3 and down-regulating Bcl-2, thereby exerting functional roles in sepsis-induced myocardial depression [36].

Furthermore, to confirm the relationship between PVT1 and the MAPK/NF-κB pathway, we added a specific inhibitor of MAPK, U0126 to _ox_LDL-induced VSMCs after PVT1 overexpression. NF-κB is considered as a pro-atherogenic factor, mainly due to its regulatory effect on pro-inflammatory proteins linked to AS, thus NF-κB inhibition could attenuate the pathogenesis of AS [37]. The LOX-1/p38 MAPK pathway participates in endothelial dysfunction in AS [38]. PVT1 was upregulated in the myocardial tissues of sepsis rats, activated the MAPK/NF-κB pathway, and thus inhibited cardiac function and promoted the secretion of inflammatory factors [18]. A previous study verified that another lncRNA, MALAT1 enhances _ox_LDL-induced autophagy of macrophages in AS by diminishing the expressions of MAPK and NF-κB in AS [39]. We finally came to a conclusion that the protective role of siRNA induced lncRNA PVT1 suppression in AS was achieved via the downregulation of MAPK/NF-κB pathway.

To conclude, these results illustrated that silencing PVT1 inhibited AS induced by high-fat diet in ApoE-/- mice, and limited HA-VSMC viability and proliferation and promoted apoptosis and cell cycle arrest, alleviated inflammation, and reduced liposome deposition via suppressing the MAPK/NF-κB pathway. The findings indicate that silencing lncRNA PVT1 could be a potential strategy to prevent and treat AS. LncRNA PVT1 is an independent hazard factor influencing the coronary AS [9] and is highly expressed in AS patients. The innovation of this study is that we explored the protective effect of siRNA silencing lncRNA PVT1 on anti-AS at molecular, cellular and animal levels by investigating the mechanism of lncRNA PVT1 in AS and the regulation of lncRNA PVT1 on the MAPK/NF-κB pathway, and thus providing new insights into further understanding of the AS pathology and reference for new prevention and treatment strategies for AS and AS-related diseases. Further study should be conducted to find out the possible applicable approaches for AS on the basis of the obtained results in this study.

### Limitations

This study revealed that lncRNA PVT1 participates in AS development and is associated with the MAPK/NF/kB pathway, and investigated the protective effect of siRNA silencing lncRNA PVT1 against AS at molecular, cellular and animal level. However, the MAPK/ NF/kB signaling pathway is a complex cellular pathway. The specific regulatory mechanism of lncRNA PVT1 in the MAPK/NF-κB pathway in AS remains unclear. Additionally, the *in vivo* study of PVT1 overexpression on AS and the MAPK/NF-κB pathway requires further investigation in future studies.

### Future directions

Further study should be conducted to probe into the specific regulatory mechanism of lncRNA PVT1 in the MAPK/NF-κB pathway in AS, the *in vivo* study of PVT1 overexpression on AS and the MAPK/NF-κB pathway, and possible applicable approach for AS based on results obtained from this study.

## MATERIALS AND METHODS

### Ethical statement

This study was carried out under the approval and supervision of the ethics committee of the Second Hospital of Hebei Medical University. All participants were informed of the study and signed the written consent. Major efforts were made to reduce the animals used and individual suffering.

### Sample collection

Totally 52 serum samples from AS patients who received diagnosis and treatment in the Second Hospital of Hebei Medical University and 38 serum samples from healthy males (aged 38~74 years old; average age: 59.5 ± 10.4) from April 2017 to September 2018 were enrolled. Inclusion criteria were: (a) all patients were diagnosed with AS, which was confirmed by measuring various indicators; (b) no patients received any treatment; (c) all patients possessed complete clinical data. Patients were excluded if they were complicated with chronic system diseases or other malignancies.

### Construction of human recombinant PVT1 lentivirus vector and small interfering RNA PVT1 lentivirus vector

The lentivirus vector pLVX-IRES-ZsGreen1 containing the green fluorescent protein (GFP) reporter gene was procured from (Clontech Laboratories, Inc., Palo Alto, CA, USA). The complementary DNA (cDNA) of human lncRNA PVT1 was synthesized by Sangon Biotech Co., Ltd. (Shanghai, China). PCR amplification was performed with cDNA as the template. The amplified PVT1 sequence was cloned into the pLVX-IRES-ZsGreen1 vector to obtain the pLVX-PVT1-IRES-ZsGreen1 plasmid, named as the lncRNA PVT1 plasmid and the empty vector pLVX-IRES-ZsGreen1.

The lentivirus-constructed siRNA LV-PVT1-RNAi-1 expression vector and hU6-MCS-Ubiquitin-EGFP-IRES-puromycin as the non-targeting control lentiviral vector were synthesized by Genechem (Shanghai, China), and recorded as si-PVT1 and si-negative control (NC), respectively.

### Cell culture

Human arterial vascular smooth muscle cells (HA-VSMCs; Shanghai Institutes for Biological Sciences, the Chinese Academy of Sciences, Shanghai, China) were cultured for 48 h in the Roswell Park Memorial Institute (RPMI)-1640 medium (Gibco Company, Grand Island, NY, USA) containing 10% fetal bovine serum (FBS) and F-12K medium (Gibco Company) at 37° C and 5% CO2. Upon 80%~90% confluence, cells were detached using 0.025% trypsin (Gibco Company) and passaged.

### Cell transfection and grouping

Well-grown HA-VSMCs were seeded in 6-well plates at the density of 5 × 106 cells/well and then assigned into 8 groups: 1) oxLDL group [HA-VSMCs were induced by 10 μg/mL (final concentration) oxidative low density lipoprotein (oxLDL; AY-1501, AngYuBio, Shanghai, China) and cultured for 48 h], 2) control group (HA-VSMCs were cultured with an equal amount of normal saline for 48 h), 3) oxLDL + si-NC group (oxLDL-induced HA-VSMCs were cultured for 48 h after transfecting with si-NC using Lipofectamine 3000 kit), 4) oxLDL + si-PVT1 group (oxLDL-induced HA-VSMCs were cultured for 48 h after transfecting with si-PVT1), 5) oxLDL + oe-NC group (oxLDL-induced HA-VSMCs were cultured for 48 h after transfecting with oe-NC), 6) oxLDL + oe-PVT1 group (oxLDL-induced HA-VSMCs were cultured for 48 h after transfecting with oe-PVT1), 7) oxLDL + oe-PVT1 + U0126 group (oxLDL-induced HA-VSMCs were cultured for 48 h after transfecting with oe-PVT1 and adding 10 ng/mL MAPK-specific inhibitor U0126), and 8) oxLDL + oe-PVT1 + DMSO group (oxLDL-induced HA-VSMCs were cultured for 48 h after transfecting with oe-PVT1 and adding 10 ng/mL DMSO).

### Establishment of AS mouse model

Specific pathogen-free (SPF) male apolipoprotein E knockout (ApoE-/-) mice in pure C57BL/6 background and wild C57BL/6 mice (weighing 20~30 g, aged 8~10 weeks; Beijing Vital River Laboratory Animal Technology Co., Ltd, Beijing, China) were fed in a SPF animal laboratory with 50~60% humidity at 22~25° C. After mice were adaptively fed for 7 days, mice were assigned in 4 groups (N = 8): 1) the normal control group (wild C57BL/6 mice were fed with conventional diet (5% fat) for 10 weeks), 2) the AS group [ApoE-/- mice were fed with high-fat diet (21% fat, 0.15% cholesterol) for 10 weeks], 3) the si-NC group (ApoE-/- mice were immediately injected with 75 μg/kg si-NC via the tail vein after feeding with high-fat diet for 10 weeks), and 4) the si-PVT1 group (ApoE-/- mice were immediately injected with 75 μg/kg si-PVT1 after feeding with high-fat diet for 10 weeks). Mice received two injections in the 1st week and then one injection per week for 3 weeks. The mice were executed afterwards.

One day before the mice were executed, no water or food was provided. The serum of 8 mice in each group was extracted from the orbital vein for enzyme-linked immunosorbent assay (ELISA). Five mice were selected from each group and the distal portions of the ascending aortas, aortic arches, and descending aortas down to the iliac bifurcations were extracted for oil red O staining. The aortas of the rest three mice in each group were used for immunohistochemistry.

### ELISA

The levels of tumor necrosis factor-α (TNF-α), interleukin (IL)-6, IL-1β, total cholesterol (TC), total triglyceride (TG), low density lipoprotein (LDL) and high density lipoprotein (HDL) were measured using IL-6 ELISA kit (IB-E10049, IBIO, Shenzhen, China), IL-1β ELISA kit (SEA563Ov03, SHRBIO, Nanjing, China), TNF-α ELISA kit (BEK-2101-1P, Biosensis, Termecula, CA, USA), TG ELISA kit (CEB687Ge02, SHRBIO), TC ELISA kit (1531142776, Jianglai, Shanghai, China), HDL ELISA kit (SEB006Hu03, SHRBIO) and LDL ELISA kit (E03944, WKSUBIO, Shanghai, China) in strict compliance with the instructions.

### Immunohistochemistry

The extracted arterial canals of mice were routinely embedded in paraffin and dewaxed. Each section was sliced into five equal parts. Sections were rinsed with phosphate-buffered saline (PBS) thrice and instilled with 3% H2O2 before maintaining for 15 min at room temperature. After 3 PBS rinses, the sections were instilled with normal goat serum blocking fluid, and maintained for 15 min at room temperature. Afterwards, the sections were incubated at 4° C overnight with 50 μL rabbit anti-human CD68 antibody (1:8,000; ab213363; Abcam Inc., Cambridge, MA, USA). After 3 PBS rinses, the sections were incubated at 37° C for 15 min with secondary antibody. Following 3 PBS rinses, the sections were instilled with 40 μL horseradish peroxidase-labeled streptavidin-working solution, followed by 15-min incubation at 37° C. The sections were again rinsed with PBS thrice, visualized with 2, 4-diaminobutyric acid, and counterstained with hematoxylin for 30 s after rinsing with distilled water, and sealed with neutral gum after dehydrating. Next, 5 visual fields with no overlaps in each section were selected to be observed under the microscope. The cells with brown-yellow- or brown-granules-stained nucleus represented CD68-positive cells. Meanwhile, 5 regions in each section were randomly selected to calculate the positive cells expressing CD68 protein.

### Oil red O staining

After mouse euthanasia, the distal portions of ascending aortas, aortic arches, and the distal parts from descending aortas down to the iliac bifurcations of mice in each group were extracted and laid flat on the white wax surface. Then, the samples were fixed with 10% (volume/volume) buffered formalin solution overnight, stained for 1 h with freshly-prepared filtered oil red O solution, and then rinsed twice with 78% methanol. The tissues were subsequently fixed on the slides and scanned with ScanScope slide scanning system (Nikon, Melville, NY, USA). Finally, the entire surface areas and oil red O-positive atherosclerotic lesion areas were scanned using Sigma Scan Pro software (SPSS Science, Chicago, IL, USA).

Well-grown cells at the logarithmic growth phase were cultured in the 6-well culture plates at 1 × 106 cells/well. When cell confluence reached 70%, the cells were rinsed thrice with PBS, fixed for 1 min with 50% isopropanol, and stained for 10 min with oil red O staining solution. Following 5-min hematoxylin staining, the red cells were observed and counted under the microscope to calculate the ratio of oil red O staining areas.

### Flow cytometry

After PBS washing and 0.025% trypsin digestion, the cells in good growing conditions were treated for 24 h at -20° C with 70% methanol (v/v) and then treated for 20 min with 50 μg/mL propidium iodide (PI) containing 10 μg/mL RNase. Finally, the flow cytometer was used to detect cell cycle distribution.

After addition with 5 μL Annexin V-fluorescein isothiocyanate and PI, the cell suspension was cultured for 10 min in the dark. Cell apoptosis was then detected after staining using the flow cytometer.

### 5-Ethynyl-2’-deoxyuridine (EdU) assay

The DNA replication ability of the well-grown cells was detected by a Cell-light EdU fluorescence kit (RiboBio, Guangdong, China). The cells were treated according to the provided instructions of the EdU kit. Five visual fields were randomly photographed under a FSX100 fluorescence microscope (Olympus Optical Co., Ltd, Tokyo, Japan). Blue fluorescence indicated all cells, whereas red fluorescence represented the replicating positive cells infiltrated by EdU. The percent of EdU-positive cells was then computed.

### Colony formation assay

HA-VSMCs of the P3 generation in good conditions were treated with 0.025% trypsin and then seeded in 6-well plates at 1000 cells/well. After 14-day culture at 37° C with 5% CO2, the cells were treated for 30 min with 75% methanol, then stained with 0.2% crystal violet and then the number of clones was counted. Each experiment was independently conducted 3 times.

### 3-(4, 5-dimethylthiazol-2-yl)-2, 5-diphenyltetrazolium bromide (MTT) assay

The cells were digested by trypsin and made into single-cell suspension at the concentration of 1 × 104 cells/mL by RPMI 1640 medium supplemented with 10% FBS. After mixing, 200 μL single-cell suspension (2 × 103 cells/well) were supplemented into each well of the 96-well plate. The blank wells were left for zero setting, and were added only culture medium without cells, serving as controls. Five duplicated wells were set up in each group. Following inoculation, the cells were cultured for 0~7 days in an incubator (37° C, 5% CO2). After 0, 24, 48 and 72 h of culture, 20 μL 5 mg/mL MTT solution was added to each well, and cultured again for 4 h before discarding the supernatant. Afterward, 200 μL dimethyl sulfoxide was supplemented to each well, followed by 10-min vibration so that crystals were fully dissolved. The optical value at 490 nm was detected.

### Quantitative real-time polymerase chain reaction (qRT-PCR)

The total RNA was obtained from cells and clinical samples using the RNAiso Plus (TaKaRa, Otsu, Shiga, Japan) and Trizol LS Reagent (TaKaRa), respectively. Then the reliability of the obtained RNA was verified by formaldehyde denaturation electrophoresis. Reverse transcription was performed using the PrimeScript™ RT Reagent Kit (TaKaRa) based on the instructions. The quantification of mRNA expressions was performed by standard real-time-PCR protocol with SYBR Premix Ex Taq (TaKaRa). Glycer aldehyde-3-phosphate dehydrogenase (GAPDH) served as a reference gene and the primers are exhibited in Table 1.

**Table 1:** Primer sequences for qRT-PCR.

**Primer**	**Sequence**
lncRNA PVT1-F (human)	CCTGTCACTCCTGCTGTGAA
lncRNA PVT1-R (human)	ACCCCCTTTACCTGCTCACT
lncRNA PVT1-F (mouse)	CCCATAACACACGCAATGAG
lncRNA PVT1-R (mouse)	TGTTGCATGTGGCATTACCT
MAPK-F	GACGAATGGAAGAGCCTGAC
MAPK-R	AGATACATGGACAAACGGACA
NF-κB-F	TGGGGACTACGACCTGAATG
NF-κB-R	GGGGGCACGATTGTCAAAGA
MMP-2-F	GTTCCCCTTCTTGTTCAATG
MMP-2 -R	CTTGCCATCCTTCTCAAAGT
MMP-9-F	CGCAGACATCGTCATCCAGT
MMP-9-R	AAGGTCAAGACGTGCCAGAG
TIMP-1-F	CCAGCGTTATGAGATCAAGA
TIMP-1-R	AGTATCCGCAGACACTCTCC
CRP	AGACATGTCGAGGAAGGCTTTT
CRP	TCGAGGACAGTTCCGTGTAGAA
GADPH-F	ACAGTCAGCCGCATCTTCTT
GADPH-R	GACAAGCTTCCCGTTCTCAG

Note: qRT-PCR, quantitative real-time polymerase chain reaction; lncRNA PVT1, long non-coding RNA plasmacytoma variant translocation 1; MAPK, mitogen-activated protein kinase; NF-κB, Nuclear factor-kappa B; MMP, matrix metalloproteinase; TIMP-1, tissue inhibitor of metalloproteinase-1; CRP, C reactive protein; GAPDH, glyceraldehyde-3-phosphate dehydrogenase; F, forward; R, reversed.

### Western blot analysis

The total protein was extracted using radioimmunoprecipitation assay lysis buffer containing phenylmethylsulfonyl fluoride (Beyotime, Shanghai, China) and protein levels in the serum were determined using the bicinchoninic acid kit. Next, an equal amount (50 mg) of protein was loaded into 10% sodium dodecyl sulfate polyacrylamide gel electrophoresis, and then moved onto polyvinylidene fluoride (PVDF) membranes (Millipore, Billerica, MA, USA) which were subsequently incubated at room temperature with tris-buffered saline tween (Boster, Wuhan, Hubei, China) containing 5% skim milk for the prevention of nonspecific binding. Afterward, the membranes were incubated at 4° C overnight with primary antibodies (all from Abcam) (Table 2), and subsequently with rabbit anti-rat secondary antibody for 1 h at room temperature. The protein was developed with an enhanced chemiluminescence reagent and visualized with a BioSpectrum gel imaging system (Bio-Rad, Hercules, CA, USA).

**Table 2:** Antibodies used in the western blot analysis.

**Antibodies**	**Item no.**	**Dilution ratio**
MMP-2	ab37150	1: 500
MMP-9	ab38898	1: 1000
CRP	ab211631	1: 1000
TIMP-1	ab234662	1: 5000
MAPK	ab185145	1: 1000
NF-κB	ab32360	1: 5000
Cleaved caspase-3	ab2302	1: 50
Cleaved caspase-9	ab232	1: 50
Cleaved PARP	ab32064	1: 5000
β-actin	ab179467	1: 5000

Note: MMP, matrix metalloproteinase; CRP, C reactive protein; TIMP-1, tissue inhibitor of metalloproteinase-1; MAPK, mitogen-activated protein kinase; NF-κB, Nuclear factor-kappa B; PARP, poly(ADP-ribose) polymerase.

### Statistical analysis

SPSS 21.0 (IBM Corp., Armonk, NY, USA) was used to process data. The normal distribution of data was verified by Kolmogorov-Smirnov test. The results were depicted in the form of mean ± standard deviation. Comparisons between two groups were analyzed using the *t* test, and comparisons among multi groups using the 1-way analysis of variance (ANOVA), followed by Tukey's multiple comparisons test. The counting data were compared using the Fisher's exact test. The *p*-value was procured by a two-tailed test and *p* < 0.05 was indicative of significant difference.

### Data availability statement

All the data generated or analyzed during this study are included in this published article.
